# Designing a Carbohydrate Counting App for Young Adults With Type 1 Diabetes: Usability Testing Interview Study

**DOI:** 10.2196/86024

**Published:** 2026-03-31

**Authors:** Asmaa Housni, Aidan Shulkin, Alexandra Katz, Giuliana Giannini, Amélie Roy-Fleming, Meranda Nakhla, Courtney South, Anne-Sophie Brazeau

**Affiliations:** 1 School of Human Nutrition, McGill University, 21,111 Lakeshore drive, Montreal, QC, H9V 1X9, Canada, 1 514-398-7848; 2 Faculté de médecine, Université de Montréal, Montreal, QC, Canada; 3 Internal Medicine, McGill University, Montreal, QC, Canada; 4 Division of Endocrinology, Montreal Children's Hospital, Montreal, QC, Canada

**Keywords:** type 1 diabetes, carbohydrate counting, mobile apps, youth, usability testing

## Abstract

**Background:**

Carbohydrate counting (CC) assists people with type 1 diabetes (T1D) adjust mealtime insulin doses; however, it is often burdensome. Mobile apps can simplify this process by automating carbohydrate estimation and insulin calculations, yet no comprehensive solution currently combines photo-based carbohydrate recognition with an integrated bolus calculator.

**Objective:**

This study aimed to identify user-informed design principles from usability testing interviews to optimize a novel app supporting young adults with T1D in CC and insulin dosing.

**Methods:**

We conducted 4 iterative rounds of usability testing interviews, each with 3 to 5 participants, using a think-aloud protocol to evaluate how easily and effectively users interacted with the app and to identify areas for improvement. Interviews were analyzed qualitatively to derive main design principles, and findings from each round informed the refinement of the app prior to subsequent testing.

**Results:**

A total of 18 participants completed the usability testing (median age of 23, IQR 19-24 y and diabetes duration of 9, IQR 6-12 y; n=12, 66.7% young women). Thematic analysis highlighted that a person-centered design that prioritizes the lived experiences of youth with T1D was essential to position the app as a self-management support system, beyond a clinical tool. Personalization was central, including customizable treatment profiles, tailored dashboard metrics, esthetic preferences, and artificial intelligence–driven recommendations based on personal trends. Early usability barriers revealed the need for intuitive navigation, streamlined multistep processes, and clear guidance for data entry and interpretation. Participants valued culturally inclusive content and familiar terminology to enhance accessibility and engagement. Users perceived strong potential for the app to centralize diabetes management tasks, integrate contextual factors (eg, exercise, diet, and timing of insulin) with glucose data, generate sharable reports to facilitate patient-practitioner communication, and strengthen self-efficacy through personalized trend analysis. Concerns about over-reliance on automation underscored the necessity of transparent data verification and user override options to maintain trust in insulin dosing decisions.

**Conclusions:**

Iterative usability testing highlighted the importance of balancing automation with user control, personalization, and contextual understanding of personal trends, as key design principles to enhance engagement and the apps’ relevance as a self-management tool. Incorporating these features into a CC and insulin-dosing app could improve self-efficacy in youth living with T1D.

## Introduction

Type 1 diabetes (T1D) is an autoimmune condition resulting in pancreatic β cell destruction [1]. People with T1D need daily administration of insulin to regulate glucose levels and prevent acute events and long-term health risks [2,3]. Intensive insulin therapy through carbohydrate counting (CC) is the recommended treatment for individuals living with T1D due to its association with lowered glycated hemoglobin (HbA_1c_) levels and the prevention of diabetes-associated complications [4-6]. Moreover, CC offers dietary flexibility by allowing individuals to tailor their mealtime insulin based on the carbohydrate content of their meals [7]. However, CC adherence can be burdensome and challenging as it requires tracking intake, reading and interpreting nutrition labels, and weighing portions [8,9]. Additionally, calculations are multifactorial and prone to error, due to the complexities of combining insulin sensitivity factor, insulin-to-carbohydrate ratio, and glucose targets [10].

The transition from childhood to adulthood introduces an additional barrier to CC, namely due to social and developmental changes commonly observed in this age group [11-13], and CC tasks transferred from parent to child. A study by Gurmani et al [14] reported that 43.6% of adolescents inaccurately calculated carbohydrates by 10 to 20 g from the actual amount. Incorrectly identified meals either lacked nutrition labels or required fiber subtraction to calculate carbohydrates accurately, underscoring the difficulty young adults face estimating carbohydrates in unpackaged foods [14]. This contributes to the broader challenge of achieving glycemic stability during adolescence and young adulthood, as evidenced by the higher HbA_1c_ levels observed in these age groups compared to others [13]. Given the various benefits of CC, strategies are needed to promote its proper use among youth.

A promising solution lies in mobile health (mHealth), a branch of telemedicine that uses technology such as mobile devices and tablets to improve health care services for both patients and health care providers [15]. In the context of diabetes management, mHealth can automate CC as a novel care approach for individuals living with T1D. In fact, the efficacy of mHealth, that is, the use of smartphone or tablet apps in improving diabetes self-management among adults, is well established [16-18]. Despite the large variety of apps able to offer photo recognition powered by artificial intelligence (AI) to quantify meal composition, most are not designed for diabetes management, few have been tested in a clinical setting, and even fewer limit their search to validated food databases only [19]. Additionally, while there are separate apps available for bolus insulin calculations, few have undergone usability testing or been developed using user-centered design approach, raising concerns about the safety of insulin calculation apps [20]. A needs assessment survey was conducted across Canada to identify the barriers and needs related to CC in people with T1D, including youth. The survey emphasized that existing mobile apps currently used for CC often fail to meet the needs of this population. A novel app incorporating features such as photo recognition, reliable nutrient values, and personalized bolus calculations could help reduce the burden of CC [21].

Scientific literature has demonstrated a need for a comprehensive app tailored for individuals living with T1D that integrates accurate CC and bolus insulin calculations. Thus, we collaborated with Ikigai Development, Inc, a company specializing in app development, to design the Petit Cactus app*,* which involved users in the development process to promote a user-centered design [22]. The Petit Cactus app was designed to include four main features: (1) the ability to calculate the number of carbohydrates in meals either through AI-powered photo recognition or through manual selection of food items, with data derived from validated Canadian food databases; (2) bolus insulin dose calculation that incorporates multiple variables (glucose levels, insulin-to-carbohydrates ratio, and correction factors), and integrates continuous glucose monitoring (CGM) data to help users visualize how logged events (ie, meals, stress, sick days, physical activities) affect glucose levels; (3) an electronic food journal for reviewing logged meals and event history; and (4) a connecting portal to a health care provider interface.

A primary objective was to identify user-informed design principles that could guide the development of this and future digital tools for supporting self-management in adolescents and young adults with T1D. Building on these principles, a broader objective was to collaboratively optimize the app’s usability to ensure it effectively meets the needs of this population.

## Methods

### Study Design

**Figure 1.**
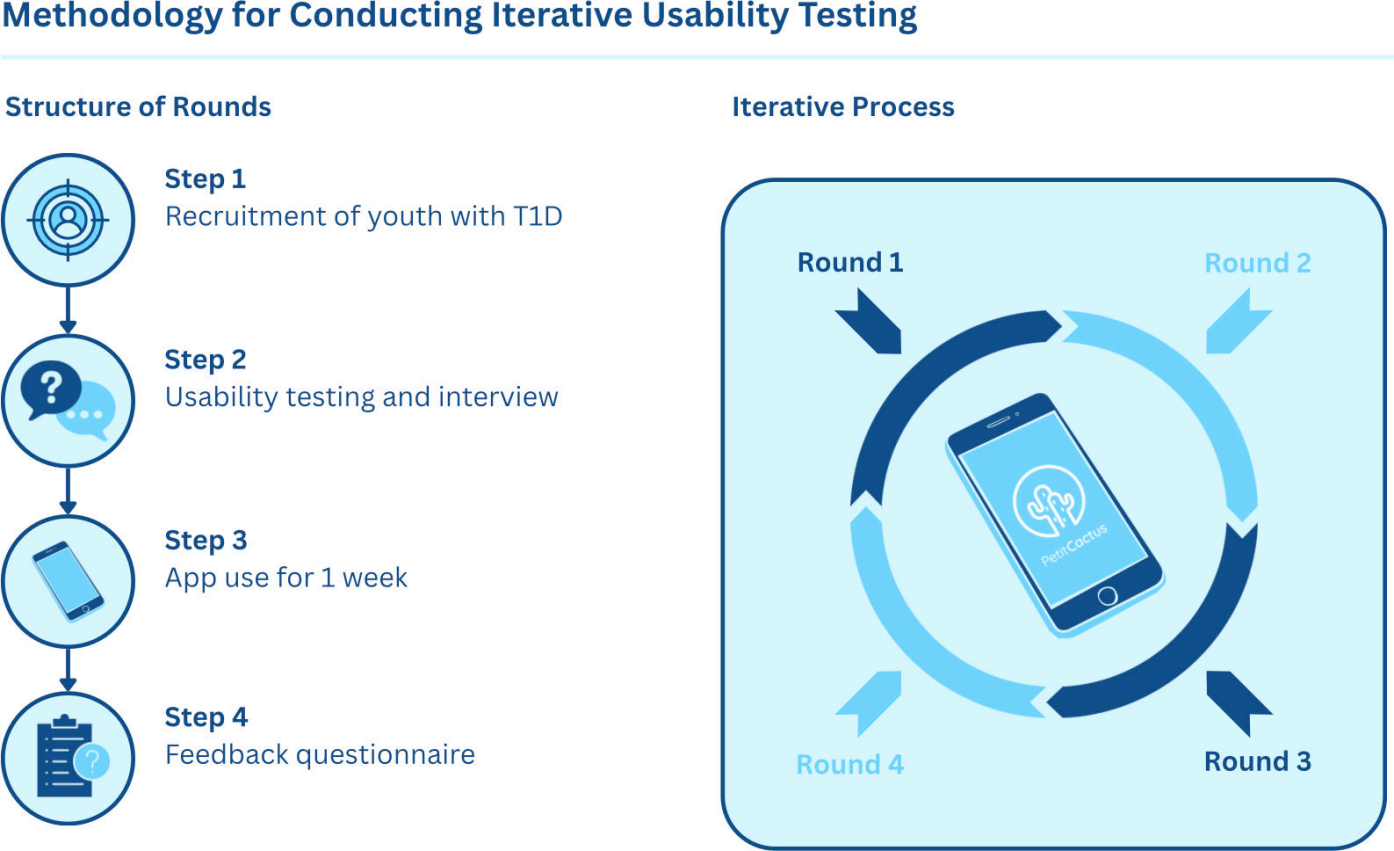
Methodology for conducting iterative usability testing. T1D: type 1 diabetes.

The study adopts a user-centered design framework, using iterative usability testing with young adults living with T1D [23]. User feedback was systematically collected and integrated into successive stages of app development to inform design refinements and enhance overall usability. We conducted usability testing to evaluate how users interacted with the Petit Cactus app and to identify usability issues, preferences, and unmet needs. Participants completed task-based interactions with the prototype while verbalizing their thoughts using a think-aloud approach, following a semistructured interview. Usability testing with young adults involved 4 iterative rounds, each with a new group of 3 to 5 participants testing an updated version of the Petit Cactus app refined based on feedback from previous rounds. The usability testing interviews were followed by 1 week of independent use and completion of a feedback questionnaire to capture additional insights (Figure 1). Data from observations and questionnaires were analyzed using inductive qualitative thematic analysis to derive user needs and design insights.

### Eligibility Criteria

Young adults aged 14 to 24 years, with a T1D diagnosis of more than 1 year, using intensive insulin therapy (ie, basal-bolus injections or insulin pump therapy), and residing in Canada, were eligible to participate in the usability testing.

### Participant Recruitment and Consent

Usability testing typically requires an average of 5 participants, as it was found to be generally sufficient in identifying significant issues for each version [23]. Recruitment was conducted using convenience sampling from BETTER (Behaviors, Therapies, Technologies and Hypoglycemic Risk in Type 1 Diabetes Registry), a volunteer registry of individuals living with T1D in Canada [24], Canadian diabetes organizations, social media, and word-of-mouth. Prior to enrollment in the study, each participant provided an informed consent and completed a demographic and background questionnaire on LimeSurvey (LimeSurvey GmbH) hosted on secure servers.

### Ethical Considerations

The study was approved by McGill University Research Ethics Board (#23-04-018). Written informed consent was obtained before participation. Adults aged 18 years or older provided their own consent, and parents or legal guardians provided consent for participants aged 14 to 17 years. All data collected were deidentified to ensure privacy and confidentiality and were hosted on secure McGill servers. Upon completion of the usability testing interview and questionnaire, participants were provided a CAD $50 (US $36.44) gift card of their choice from a selection of retailers as a compensation for their time and participation in the study. The study was conducted in accordance with the principles of the Declaration of Helsinki for research involving humans and/or human data.

### Procedures

Following consent, participants were contacted by email to schedule a usability interview. Subsequently, a link to a Microsoft Teams meeting was sent. One or two days preceding the interview, participants received a user guide to the app with access and log-in information. Interviews were 60 to 90 minutes in length and included the participant, the interviewer, and an observer who took notes to complement verbatim transcripts, when necessary. Prior to commencing the interview, participants were reminded of the objective of the usability testing and their role in the co-design process: to provide feedback on their impression of the app and necessary changes required to improve its functionality. Each session consisted of two components: (1) task-based usability testing and (2) a semistructured interview. During the usability testing component, participants were asked to interact with the app in a sequential order, starting with completing their user profile and dashboard, and then later trialing core features (ie, meal entry and the bolus insulin calculator; Multimedia Appendix 1). Using a think-aloud approach, participants verbalized their thoughts while completing predefined tasks. Probing questions from the usability guide were used as needed to clarify navigation challenges, functionality, design, and ease of use. Following task completion, a semistructured interview was conducted to explore participants’ broader perceptions of the app, expectations, and experiences using the app. Following the interview, participants used the app for one week and were then requested to complete a final questionnaire including 8 open-ended questions. The questionnaire was developed by the research team to better understand potential barriers to app use, the usability of core features (ie, the food journal, meal entry and CC, readability of glucose graphs, desired support from health care providers), motivations for trying the app, and suggestions to enhance engagement (Multimedia Appendix 2). The open-ended format was selected to allow participants to elaborate on their experiences and identify unanticipated issues.

### Data Collection

The interviews were recorded and transcribed using Microsoft Teams. Three members of the research team (AH, AS, and GG) verified the accuracy of the transcripts and anonymized all identifiable information. The transcripts were then translated from French to English using a forward-backward translation method [25], when necessary by AH and verified by GG and AS, all of whom are fully bilingual.

### Data Analysis

The transcripts were open coded independently by 3 researchers (AH, AS, and GG), using MAXQDA (version 24; VERBI GmbH), to develop a preliminary code book [26]. Interviews were conducted in 4 rounds, with thematic saturation reached within each round, defined as the point at which no new ideas emerged and no new codes were identified, and overall saturation achieved by the fourth round. Two researchers then independently coded all transcripts using the finalized codebook. To ensure consistency, 2 transcripts were initially co-coded, and discrepancies were discussed until consensus was reached, leading to refinement of code definitions. The remaining transcripts were coded independently, with regular meetings held to compare coding decisions and resolve any discrepancies through discussion. An inductive thematic analysis was conducted to interpret the data [27]. After transcripts were first coded, codes were then iteratively grouped and refined to identify themes based on patterns and similarities across the dataset.

## Results

### Participants

Twenty participants were recruited and provided consent. However, 2 participants were excluded due to not meeting the inclusion criteria for age and T1D diagnosis, despite self-reporting eligibility in the initial online survey. A total of 18 eligible participants completed the interview and the questionnaire. About two-thirds of the participants identified as women (n=12, 66.7%), and Caucasian (n=11, 61.1%). The median (IQR) for age was 23 (19-24) years and for diabetes duration was 9 (6-12) years. Almost all participants (n=17, 94.4%) used CGM systems. The majority used an insulin pump to administer insulin (n=14, 77.8%), and 12 (66.7%) used an automated insulin delivery (AID) system. Participants’ characteristics are outlined in Table 1.

**Table 1.** Participant characteristics.

Characteristics	Value
Demographics (N=18)	
Age (y), median (IQR)	23 (19-24)
Gender (women), n (%)	12 (66.7)
Country of birth (Canada), n (%)	15 (83.3)
Ethnicity^a^, n (%)	
Arab	1 (5.6)
Black (African, Afro-American, and Caribbean)	3 (16.7)
Indigenous (First Nation)	1 (5.6)
White or Caucasian	11 (61.1)
Currently studying^b^, n (%)	13 (72.2)
Highest level of education acquired, n(%)	
High school level	7 (38.9)
CEGEP^d^, vocational or community college	6 (33.3)
University level	5 (27.8)
Medical history and diabetes management (N=18)	
Diabetes duration (y), median (IQR)	9 (6-12)
Had at least 1 diabetes-related consultation in the last year with, n (%)	
Medical specialist (endocrinologist, pediatrician, or internist)	17 (94.4)
Family physician or general practitioner	15 (83.3)
Registered dietitian	12 (66.7)
Registered nurse	12 (66.7)
Use continuous glucose monitoring systems (N=18), n (%)	17 (94.4)
Dexcom G6	10 (55.6)
Freestyle Libre	1 (5.6)
Freestyle Libre 2	1 (5.6)
Guardian Sensor 3	2 (11.1)
Guardian Sensor 4	3 (16.7)
Use insulin pens or syringes exclusively (N=18), n (%)	4 (22.2)
Use an insulin pump (N=18), n (%)	14 (77.8)
Use of pump features in the last week^c^ (n=14), n (%)	
Use an integrated bolus wizard or calculator function	14 (100)
Use an automated insulin pump	12 (86)

a Two participants do not know their ethnicities.

b One participant preferred not to answer.

c Proportion among the 14 pump users only.

d CEGEP: Collège d'Enseignement Général et Professionnel (general and professional college).

### Critical Issues and User Feedback Across Rounds: Thematic Analysis

#### Overview

**Figure 2.**
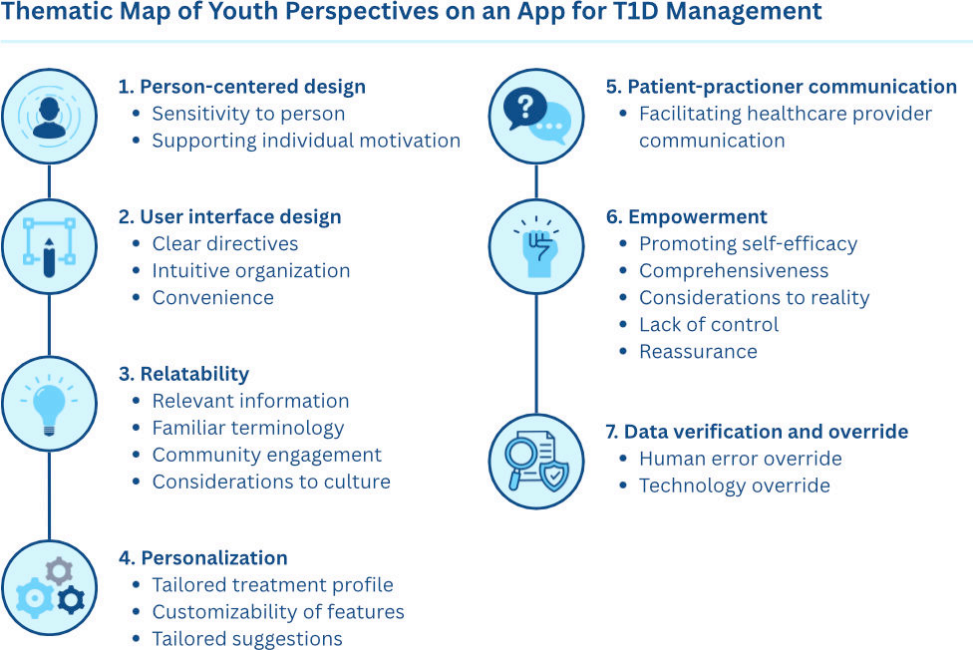
Themes and codes identified through usability testing of a type 1 diabetes (T1D) management app with youth.

Data from the usability testing of the CC app with young adults with T1D was analyzed using thematic analysis through which usability issues and user preferences identified were coded and iteratively grouped based on shared patterns across 4 rounds. This process resulted in 7 themes that represent recurring usability priorities and corresponding design principles. The codes and emerging themes, representing distinct patterns across all rounds of the dataset, are presented in Figure 2. A summary table of all themes, associated codes, and illustrative participant quotes is provided in Multimedia Appendix 3. Evaluations and suggestions for the app’s features were compiled to illustrate how the features evolved across the usability testing rounds (Table 2).

**Table 2.** Feature development through usability testing rounds.

Features	Round 1	Round 2	Round 3	Round 4
Photo recognition for carbohydrate suggestions	The photo capture feature for carbohydrate estimations was presented unclearly, leading some participants to mistake it for a tool to simply add pictures of meals.	User feedback was incorporated to improve the presentation of the photo recognition feature, which was clarified by adding prompts to explain its function. Photo uploading remained slow.	Ease of use was enhanced by adding simple options to edit food items incorrectly recognized by AI^a^.	The algorithm was optimized for accuracy, with faster photo uploading.
Meal logging	Meal logging was designed with step-by-step navigation instead of showing everything on one page. However, it was overly complex, unintuitive, and too lengthy. The food database also had issues with irrelevant items and inaccurate search results.	The food database was subsequently improved by adding a better search function that presents key food items first, while retaining a large database.	The next iteration addressed this by displaying everything on the same page with streamlined manual entry and dropdown features. The food database was refined to prioritize commonly used items, but estimating quantities remained challenging.	The final iteration enhanced functionality by allowing users to copy or edit previously logged meals, offering more units of measure and options to determine food quantities. It also included options to add notes or assign meal tags (eg, high fat, high protein, pre or post-workout, or injection timing).
Event logging	The feature was not included in this round, but users suggested it to track events other than meals that could impact glucose levels.	Initial concept presented.	The full feature was added, allowing users to track various events beyond meals, such as glucose and insulin (independent of meals), and physical activity.	More events were added, such as the menstrual cycle, medication, and a customizable event, to enable more comprehensive event tracking.
Insulin dosing calculator	Basic model for dosing recommendations.	No changes were made.	The feature now presents an added safety measure. Users are shown their glucose levels and ratios and must confirm entries before calculations.	No changes were made.
Dashboard	The dashboard featured a basic design with limited functionality, presenting statistics on the number of meals, units of insulin, and glucose levels, all compiled over the course of the day.	User feedback was incorporated, resulting in a clearer visual presentation and the addition of adjustable timeframes for compiling statistics.	The final design included a personalized data display with the addition of a graph to show glucose levels alongside logged events (meals, activity, stress, and others).	When the CGM^b^ device is connected, glucose levels are displayed in real time, and the cactus avatar dynamically changes to reflect these levels.
Profile settings	Basic settings included name, age, date of birth, gender, target glucose levels, insulin modality, glucose monitoring preferences, and app language.	Settings were expanded to enable users to enter multiple correction factors, input their basal insulin regimen, as well as the option for users to remove information irrelevant to their diabetes management.	Further refinements were made, including the option to add a profile picture or an avatar.	No changes were made.
Connection to a health care provider portal	Not available. Participants recommended a direct method to share data with the health care team.	Initial concept of provider connection presented.	The prototype was tested with basic integration, including an option to link the account with a health care team portal.	No changes were made.
AI chatbot	The AI chatbot was presented unclearly, often mistaken for a help center.	Prompts and examples of queries were added to clarify its use.	In the final iteration, the AI was integrated to provide personalized feedback based on previous discussions.	No changes were made.

a AI: artificial intelligence.

b CGM: continuous glucose monitoring.

#### Theme 1: Person-Centered Design

This theme reflects the importance of designing the app with youth living with T1D in mind, prioritizing their perspectives and lived experiences rather than focusing solely on what may be deemed important from a clinician-centered perspective. Participants consistently emphasized the need for the app to acknowledge the person living with T1D, not only the condition it aims to support. This theme evolved across the 4 rounds to reflect a progression and growing interest in emotional well-being and sensitivity to stigma within the app’s design.

Initially, participants highlighted the importance of prioritizing personal profile settings (eg, name, gender, and date of birth) before treatment-related parameters during onboarding. This approach was appreciated for making the app feel more personal.

In later rounds, participants further distinguished information relevant to health care providers from that intended for users with T1D. While clinical parameters such as blood glucose targets were considered essential during medical appointments, participants expressed a desire not to have these metrics displayed at all times. Suggestions included the ability to customize dashboard displays, such as hiding A_1c_ levels, which some found stress-inducing. They also recommended avoiding design elements that could unintentionally reinforce negative perceptions (eg, a thumbs down for late insulin administration) and replacing red and green indicators with neutral colors to classify food choices:

*Especially when you’re a young teenager and you see your A_1c_’s high, it can stress you out. Maybe it could be an option to hide it on your dashboard*.

[Woman, 20 years, 13 years living with T1D]

*Say for example I had a bad few days I wouldn’t want to remind myself of it all the time.*

[Man, 24 years, 9 years living with T1D]

The final round added features to support individual motivation. For instance, participants suggested integrating challenges and objectives that align with users’ preferences for managing their diabetes, focusing not just on what they need to do but on how they want to experience and engage with the process. This also included offering peer support and the option to send encouragement messages to others within the app, if they consent to it.

#### Theme 2: User Interface Design

During the first round, users identified foundational problems. They reported significant challenges with navigation, struggling to locate key elements such as the photo recognition feature, the dashboard, the settings and treatment profile, and the help menus. There was a lack of clear directives, leaving users uncertain about what needed to be entered and unclear about the descriptions of what was displayed. Prompts, descriptors, and visual cues were suggested to optimize the user’s comprehension of entry requirements.

By round 2, navigation improvements reduced these challenges; however, multistep processes remained cumbersome. For instance, meal logging required navigating through multiple pages (first to confirm food items recognized by the AI algorithm, then confirm the detected quantities, and finally to confirm bolus calculations), whereas users suggested a streamlined approach using a list or drop-down menu in the same page. Another important aspect is intuitive organization, ensuring that the order of information display is practical. For example, users instinctively clicked on their profile picture to navigate back to their profile.

In later rounds, critical navigation issues were resolved, and only minor refinements were suggested to improve convenience, such as faster photo upload and quicker analysis of the photos. Participants also highlighted the value of automatic logging of meals in the food journal, when a photo is used to estimate carbohydrate content, along with glucose trends and insulin dosing. They further requested simple data extraction for the food journal and the ability to filter and sort information efficiently. This was further reinforced in the feedback questionnaire (Multimedia Appendix 4).

#### Theme 3: Relatability

This theme focused on enhancing relatability by providing relevant and accessible information while minimizing complexity. In round 1, participants emphasized the need to streamline the food journal feature by avoiding overly detailed descriptions in the database and instead prioritizing a simple, comprehensive list of commonly used items. The importance of using familiar terminology was highlighted to improve user comprehension and align with user preferences (eg, correction factor vs insulin sensitivity factor, 1 cup vs 250 ml).

In later rounds, fostering community engagement became a key focus. Participants proposed adding an in-app peer messaging feature, and a *Frequently Asked Questions* section to promote peer feedback and provide actionable insights. Additionally, participants stressed the importance of cultural inclusivity by ensuring that the app’s content, such as food items in the database, is relevant and accessible to users from diverse backgrounds:

*I think that the different cultures and cultural foods might be something to take into consideration. [...] We really need to make sure there are many different people and many different cultures represented.*

[Woman, 22 years, 11 years living with T1D]

#### Theme 4: Personalization

In earlier rounds, participants emphasized the importance of tailoring the treatment profile to individual needs, including options for CGM connection, weight tracking, injection reminders, and specifying insulin treatment types (eg, pen or injections vs pump). This was particularly relevant when discussing the dashboard and the types of statistics users wanted displayed, depending on their insulin delivery methods and glucose level monitoring preferences. Beyond diabetes management, customizability of the app itself is important. Participants expressed a desire to personalize its esthetics, such as colors and design, as well as practical features like glucose units, custom tags, data visualization, and the organization of the features to align with their individual routines and preferences.

By later rounds, participants suggested the incorporation of AI technology to provide tailored diabetes management recommendations based on user trends and status:

*If, say you’ve had too many [hyperglycemic episodes] and it’s dinner time. [...] Maybe the AI could give suggestions based on your statistics for the day*.

[Man, 18 years, 12 years living with T1D]

#### Theme 5: Patient-Practitioner Communication

Participants highlighted the potential for the app to enhance patient-practitioner communication, facilitating more effective diabetes management. Initially, participants reported that these features could empower users to take charge of their management during appointments with their health care team, fostering more productive discussions:

*I went to see the dietitian, and I had to provide a dietary recall, but trying to remember everything I’d eaten, it’s definitely more tedious. But to see that [the food journal] is accessible, to have it on hand all the time, and to be able to enter it as I go along, I find it really practical*.

[Woman, 19 years, 10 years living with T1D]

By later rounds, the emphasis shifted to refining and expanding these features. Participants suggested generating downloadable reports that combine dietary data, automatically generated from photos taken with the app, with glucose levels and insulin doses, to facilitate sharing with health care professionals and supporting more informed decisions.

#### Theme 6: Empowerment

The theme of empowerment focuses on how the app can enhance users’ confidence and ability to manage T1D effectively, while addressing concerns about over-reliance on technology. In earlier rounds, participants emphasized how a CC app could empower young adults with T1D by promoting self-efficacy. The app’s ability to centralize tools and information into a single platform was highlighted as essential for simplifying diabetes management. Features like long-term tracking and trend analysis allowed users to understand factors influencing glycemia, such as meals, activity, stress, and timing of insulin injections:

*If I look at my graph later and see that one day I was trending high all day, but then I look back at the food journal and remember what I ate, I’ll understand better*.

[Woman, 19 years, 10 years since diagnosis]

Additionally, the app reassured users by validating daily behaviors, even those deviating from guidelines, providing a realistic and supportive approach to condition management:

*I really liked [the feature of inputting insulin before or after a meal]. I sometimes forget to give myself insulin before I eat. I think it’s important to see the trend.*

[Man, 18 years, 12 years living with T1D]

In later rounds, participants expressed appreciation for these empowering features but raised concerns about the potential trade-off between reliance on technology and their sense of control. Some users noted skepticism regarding the accuracy of the app’s recommendations, which they felt could undermine their self-efficacy.

Discussions emphasized the importance of building user trust to address these concerns. Participants suggested that transparent mechanisms to verify the app’s outputs and ensure accuracy would help mitigate doubts. This evolution across rounds demonstrates the balance needed between empowering users and maintaining their confidence in the app as a reliable support tool for diabetes management. This is further highlighted in the feedback questionnaire, where participants expressed reluctance to rely on AI-driven photo recognition if the carbohydrate content of meals is already known (Multimedia Appendix 4).

#### Theme 7: Data Verification and Override

Participants highlighted the importance of data verification and override capabilities within the app to ensure accurate diabetes management. In earlier rounds, the focus was on addressing critical issues, such as implementing mechanisms to override technological errors and the ability to accept or reject automated suggestions made by the app. They also stressed the importance of addressing human error by incorporating prompts that encourage users to verify and correct data entries to enhance reliability.

By later rounds, the emphasis shifted to refining these features, including streamlining the verification process to avoid unnecessary disruptions during app use. Participants suggested simplifying error notifications to ensure they were clear but not intrusive and incorporating an option to review and confirm CGM readings. These minor improvements aimed to strike a balance between maintaining user control and creating a seamless experience. 

## Discussion

### Principal Results

The usability testing revealed 7 major themes highlighting key design principles. These themes focused on young adults’ empowerment, enhancing patient-practitioner communication, data verification and override for accuracy in diabetes management, an intuitive user interface design, and the relatability of the information provided. While both the person-centered design and personalization themes reflect participants’ desire for individual relevance, they differ in scope and intent. Person-centered design represents a broader guiding principle emphasizing empathy and how the app acknowledges the person behind the condition. In contrast, personalization refers to the practical and technological implementation of this principle ie, how the app’s content, settings, and recommendations adapt to the user’s specific needs and data.

### Comparison With Prior Work

Clinical guidelines recommend adjusting meal-time insulin dose to manage glucose variability due to different meal compositions [28,29]. However, there is no consensus as to which insulin strategies to use. Significant interindividual differences in insulin dose requirements exist, necessitating personalized advice based on postprandial glucose monitoring for several hours afterwards [30]. This is consistent with a major emerging theme: empowering users to gain control over their T1D management by understanding personal trends and factors affecting glycemia. While users appreciated receiving personalized AI-generated recommendations based on their trends, the sense of empowerment mainly came from accessing personal statistics and tracking various factors.

The interest to learn about personal trends aligned with participants’ desire to remain in control and avoid full reliance on the app, which could hinder self-efficacy. Some users were skeptical about the accuracy of AI-generated information, emphasizing the need for safety mechanisms for data verification and the ability to override when necessary. This concern aligns with previous research on the lag between interstitial and capillary glucose levels, which led to data distrust and prompted some CGM users to rely on capillary glucose to clarify high and low readings [31]. Similarly, a recent study examining patient perspectives on AI in health care highlighted varying levels of comfort depending on the role of AI in care, with higher acceptance of apps that play an assistive role (eg, digital scribing or radiology review), and lower comfort when AI systems directly influenced decision-making [32]. Together, these findings highlight the tension between empowerment and over-reliance on technology in contexts where trust in automated systems is still evolving.

This sense of empowerment was further emphasized in the context of patient-practitioner communication. Participants reported that access to their own data enabled them to take a more active role in diabetes management by sharing relevant information and tracking topics they wished to discuss during medical appointments. This form of empowerment is particularly important during adolescence, a time when young people are striving to gain independence and shape their personal and social identities [33]. It is especially relevant given evidence that young adults with T1D often report health care needs and priorities that significantly differ from those perceived by primary caregivers and health care professionals [34]. Consistent with literature on shared medical appointments, which emphasize patient leadership and active participation in care, greater access to and understanding of personal health data has been shown to enhance patients’ ability to engage more confidently in discussions with clinicians, supporting more collaborative and patient-led care [35].

Importantly, access to information must be implemented through a person-centered lens to balance what information youth want to access and *how* they want to engage with it. For example, participants did not want glucose data displayed on their dashboard each time they opened the app. It is well documented in the literature that living with and managing T1D is associated with significant psychological burden, with young people at increased risk of psychiatric disorders compared to peers without diabetes [36]. More specifically, data overload and constant exposure to CGM and insulin pump data have been identified as sources of stress [37]. Notably, while CGM use has been associated with improved well-being among young people, only a minority report regularly engaging in detailed data analysis [38]. These findings highlight the importance of selectively presenting information in digital tools intended to support diabetes management. At the individual level, participants voiced different needs: some valued a comprehensive app that consolidates all information necessary for diabetes management and reduces the need to juggle multiple sources, while others felt that repeatedly displaying data such as glucose readings or insulin doses was redundant, given its availability through CGM and insulin pump reports. Filtering information, by suppressing, deleting, or selecting irrelevant information, has been identified as a helpful strategy for mitigating information overload [39]. This aligns with personalization as a key design principle, enabling users to determine which factors to track, how data are visualized, and which trends are emphasized.

Participants also expressed a desire to access consolidated data, with logged meals alongside corresponding glucose values, to better understand personal patterns. The multifactorial nature of glycemic management is well established, as insulin requirements are influenced by factors beyond carbohydrate intake, including physical activity, stress, and illness [40]. This complexity highlights the importance of tools that can help support decision-making in diabetes management. Consistent with this, findings from a needs assessment survey emphasized the importance of tracking factors beyond dietary intake, such as hormonal changes, timing of insulin delivery, and sleep, alongside glucose data [21].

These considerations are particularly important given clinical appointments often prioritize glucose data and glycemic management, which, although essential, may leave limited space to address broader psychosocial, behavioral, and contextual factors that shape day-to-day management, largely due to time constraints [41]. Designing the app around youths’ lived experiences, rather than solely around clinical priorities, helps bridge this gap and ensures greater relevance and usability. Incorporating features that reflect real-world practices, such as tracking whether insulin was administered before or after meals despite guidelines recommending injecting before meals [42], acknowledges daily realities and avoids setting unrealistic expectations. Consistent with this, a study on the co-design of a digital mental health platform for young adults highlighted the importance of relatability, with participants emphasizing that the platform should be one they can identify with to support meaningful engagement [43].

### Strengths and Limitations

These results should be interpreted in light of the study’s limitations. A major limitation is that most participants were users of AID, relying on systems that automate insulin delivery and integrate CGM data. Consequently, feedback may under-represent the perceived value of features aimed at supporting manual bolus calculations, while overrepresenting preferences for features that support retrospective reflection and learning from personal trends rather than real-time decision support. In addition, AID users may represent a subgroup with higher baseline engagement with diabetes technologies, greater digital literacy, and increased comfort with data interpretation. While some app functions may overlap with AID features, the app serves a complementary rather than redundant role. AID systems focus on insulin automation but provide limited insight into the behavioral and contextual factors influencing glucose stability. In contrast, the proposed app supports self-awareness, education, and behavioral reflection by linking glucose trends with meal composition, stress, physical activity, and other lifestyle factors. It also facilitates communication with health care providers through integrated reports that combine these contextual data. Finally, its relevance extends to individuals on basal-bolus injection therapy, who continue to make daily dosing decisions manually, and to those without access to AID systems due to cost, eligibility, or access limitations. Other limitations include the potential selection bias arising from the nonrandom sample, which may affect generalizability. However, the users represented various ethnicities, genders, education levels, and insulin treatment modalities. Researcher bias was mitigated by using the same interviewer throughout, ensuring consistency in questioning style, tone, and data collection procedures across rounds. In addition to having an observer present during the interviews, subjectivity in data analysis was addressed by having 3 coders reach consensus through discussions. Observer bias was minimized by adhering to a preset questionnaire and having no prior relationships with participants. Due to the potential for authority and response bias, the interviewer mitigated their impact by establishing rapport with participants to create a comfortable and nonjudgmental environment, used neutral language and tone to avoid perceived authority, and encouraged openness by reassuring participants that their honest opinions were valued.

A key strength of the study is that end user needs directly informed the app’s development, with participant input integrated iteratively across all design rounds. This co-design approach ensured that the resulting features were grounded in lived experience and reflected the priorities of young adults with T1D. By continuously refining the app based on user feedback, the design process enhanced relevance and usability, laying a strong foundation for future pilot testing and real-world engagement.

### Conclusions

Petit Cactus is a mobile app that enables carbohydrate estimations through photo recognition and supports automated insulin dosing within a single, user-informed platform. Through its co-design process, the study identified design principles that extend beyond this specific application, emphasizing person-centered, intuitive, and emotionally sensitive interfaces that align with users’ daily routines and language. Participants also underscored the importance of personalization, data accuracy, and effective communication tools to support meaningful interaction with health care providers. These insights could inform the design of digital health tools aimed at supporting diabetes self-management. Usability testing has refined Petit Cactus accordingly, and a pilot trial will next evaluate its impact on engagement and glycemic outcomes among young adults with T1D.

## Supplementary material

10.2196/86024 Multimedia Appendix 1 Interview guide and questions.

10.2196/86024 Multimedia Appendix 2 Feedback questionnaire.

10.2196/86024 Multimedia Appendix 3 Themes and codes description

10.2196/86024 Multimedia Appendix 4 Appreciations and suggestions from the feedback questionnaire after 1 week of app use.

## Abbreviations:

AI: artificial intelligence
AID: automated insulin delivery
BETTER: Behaviors, Therapies, Technologies and Hypoglycemic Risk in Type 1 Diabetes Registry
CC: carbohydrate counting
CGM: continuous glucose monitoring
HbA_1c_: glycated hemoglobin
mHealth: mobile health
T1D: type 1 diabetes
