# Supplementary material for: Designing a Carbohydrate Counting App for Young Adults With Type 1 Diabetes: Usability Testing Interview Study
Source: J Med Internet Res. 2026 Mar 31;28:e86024. doi: 10.2196/86024 (PMC13037768; doi:10.2196/86024)
Supplement: Multimedia Appendix 1 [file jmir-v28-e86024-s001.docx]

**Multimedia Appendix 1 - Interview guide and questions.**

I. Tasks requested during the interview:

- Complete your profile setting

- Enter a meal

- Calculate the insulin bolus dose

- Consult the food journal

- Consult the dashboard

- Read the glucose level and event graph

- Ask a question to the AI chatbot

II. Probing questions:

- What was the user experience like in using the app to perform this task?

- Was it easy or difficult to navigate this task?

- What do you think of the design of the steps to follow?

- Why did you navigate to [Page A] instead of [Page B]?

- What prompted you to click on [a specific interaction]?

III. General concluding question:

- What features are most important to you?

- What features are least important to you?

- What features were you expecting to find but did not?

- What features did not work as expected?
