# Supplementary material for: Designing a Carbohydrate Counting App for Young Adults With Type 1 Diabetes: Usability Testing Interview Study
Source: J Med Internet Res. 2026 Mar 31;28:e86024. doi: 10.2196/86024 (PMC13037768; doi:10.2196/86024)
Supplement: Multimedia Appendix 2 [file jmir-v28-e86024-s002.docx]

**Multimedia Appendix 2 - Feedback questionnaire.**

1. What factors would make you not reuse this application?
2. How could the food journal be better organized?
3. If applicable, which parts made meal entry and/or carbohydrate calculation unnecessarily complicated?
4. What would make the blood sugar level graphs easier to read?
5. What kind of support from healthcare professionals would you like to receive through the application?
6. What interested you in trying the application?
7. What features would make PetitCactus more engaging? (e.g., having characters, challenges and rankings, progress bar, maintaining a streak, earning points for groceries, travel giveaways...)
8. Have you seen other applications do something similar?
9. Please leave any other suggestions or comments you may have.
