# Supplementary material for: Designing a Carbohydrate Counting App for Young Adults With Type 1 Diabetes: Usability Testing Interview Study
Source: J Med Internet Res. 2026 Mar 31;28:e86024. doi: 10.2196/86024 (PMC13037768; doi:10.2196/86024)
Supplement: Multimedia Appendix 3 [file jmir-v28-e86024-s003.docx]

**Multimedia Appendix 3 - Themes and codes description.**

| **Themes** | **Codes** | **Description** | **Quotes examples** |
| --- | --- | --- | --- |
| Person-centred design | Sensitivity to person | Considering the impact of information on the wellbeing of the user, which involves discerning information relevant to the healthcare professional versus relevant to the user’s ability to manage T1D. Finding a balance between encouraging users to self-manage T1D all the while not discouraging or causing undue stress. | “*I like the order; how first it's your name, your gender, your birthday... And then the last thing is to put in your diabetes [information]. It’s not like ‘oh, right away, put your bolus before your name’*.”  - Woman, 20 years old, 13 years living with T1D  “*Especially when you're a young teenager and you see your A1c’s high, it can stress you out. Maybe it could be an option to hide it on your dashboard.*”  - Woman, 20 years old, 13 years living with T1D |
|  | Supporting individual motivation | Adding challenges and objectives for the app to align with how individuals prefer to engage with their diabetes management, focusing not just on what they need to do, but on how they want to experience and interact with the process. | *“Maybe add some challenges or objectives, then we could see whether we’ve reached them or not. I think it would be a good idea to involve friends [...], you can look up their profiles, see their trends, and send them encouragement, like a ‘send encouragement’ button. When you visit their profile, you can interact with them.”*  - Woman, 17 years old, 5 years living with T1D |
| User interface design | Clear directives | Prompts, descriptors and visual cues optimize user’s understanding of entry requirements. | *“I think for people who are newly diagnosed, it would be better to guide them on how to find [ratios and correction factors to enter into the app] in the pump, if there’s some kind of video or something to tell them. Because it’s very easy to find on the pump, but still for someone who’s newly diagnosed, it might be little [overwhelming]. So that would be a recommendation.”*  *- Woman,* 22 years old, 11 years living with T1D |
|  | Intuitive organization | The order in which information is displayed (i.e. app design) is practical, and the ability for users to perform intuitive behaviors (i.e. intuition to click on profile picture to return to profile) is met. | *“All the meal log steps should be on the same page [rather than navigating multiple pages], with the save button at the bottom.*  - Woman, 24 years old, 8 years living with T1D |
|  | Convenience | Ease of use of app functions including data entry (i.e. photo recognition), data extraction (i.e. insulin dose), data export (i.e. food journal), and ability to filter and sort information. | “*Overall, it seems quite easy, quite simple. For me, I love the simplicity of it, like how simple it is to navigate. Nothing too [overwhelming]. If something's overwhelming, people will just be like ‘Ok, I don't care, I don't want to do this’*.”  - Woman, 22 years old, 11 years since diagnosis. |
| Relatability | Relevant information | Prioritizing pertinent information for the user’s T1D management while minimizing exposure to unnecessary and complex information. | “*For instance [when I enter the word banana in the food item search bar], I feel like the propositions are too specific. [...] The first thing is: Dessert, pudding, banana. It doesn't offer me the fruit banana first, but it offers many things made of bananas.*”  - Woman, 23 years old, 10 years living with T1D |
|  | Familiar terminology | Considering the interchangeability T1D terminology (i.e. correction factor vs insulin sensitivity factor) and synonymous language ( 1 cup vs 250 ml) with the goal of being inclusive to all possible terminology used by users. | *“The ISF, which is the insulin sensitivity factor but is also called the correction factor. So yes, having the term correction factor as well.”*  - Woman, 22 years old, 11 years living with T1D |
|  | Community engagement | Continuous end-user collaboration and engagement for the purpose of peer support and app feedback. | *“I’d like to be able to connect and communicate with peers my age using the app.”*  - Man, 24 years old, 6 years living with T1D  “*It can be practical to have [an FAQ section]. Sometimes there are people who think they have a “silly” question but not everyone knows about it. So, if there was [an FAQ section], people would be able to get a precise answer quicker, [without feeling ashamed]*.”  - Woman, 19 years old, 10 years living with T1D |
|  | Consideration to culture | Diversifying food database to include food products from various cultures with the goal of the app being relevant to all users. | *“I think that the different cultures and cultural foods might be something to take into consideration. [...] In the future, if it’s possible to collaborate with people from different cultures, that would be helpful. In Canada, just using Western food might be a bit limiting, especially considering the different cultures even within Canada. So we really need to make sure there are many different people and many different cultures represented.”*  - Woman, 22 years old, 11 years living with T1D |
| Personalization | Tailored treatment profile | Relating to user’s ability to optimize T1D management through various features such as CGM connection, weight tracking, injection reminders, inputting insulin therapy (i.e. pen vs pump). | *“Not all of the information on glucose levels would be useful [for me], but it would maybe be useful for injection users or people with a CGM that want to have the data in this app. But I know I wouldn't use this information.”*  - Woman, 23 years old, 10 years living with T1D  *“If your glucose levels is high for a long period of time, once it goes down, you can feel [as if you’re in a hypoglycemic episode], but it may not actually be a physiological [hypoglycemic episode]. That would be nice to add [...] and make it an event to be able to associate it with a glucose reading”*  - Man, 24 years old, 9 years living with T1D |
|  | Customizability of features | Personalizing app design such as the general aesthetic, language settings, available units of measurement, custom tags, custom data visualization. | “*Since kids are using [the app] [...] if there was a way to put an avatar like a soccer ball or a flower [...] I think that would be a cute [way] for children to be able to personalize their account.*”  - Woman, 20 years old, 13 years living with T1D |
|  | Tailored suggestions | Using trends and status of user to generate diabetes-specific management suggestion through AI technology. | “*If the AI would be able to give you a suggestion… If, say you've had too many [hyperglycemic episodes] and it's dinner time. [..] Maybe the AI could give suggestions based on your statistics for the day*.”  - Man, 18 years old, 12 years living with T1D |
| Patient-practitioner communication | Facilitating healthcare professional communication | Facilitate T1D-related information transfer between patient and practitioner: assist the user to retrieve and share pertinent information from the app to healthcare professionals (i.e. # of hypoglycemia in the past 3 months), and allowing healthcare professionals to easily retrieve information on their own as well. | *“It would be useful for [the app] to tell you how many [hypoglycemic episodes] you have had. Because often, let's say when you go see your endocrinologist, they ask you how many [hypoglycemic episodes] you have had and you're like thinking in your head*.”  - Woman, 20 years old, 13 years living with T1D  “*This morning, I went to see the dietitian, and I had to provide a dietary recall, but trying to remember everything I'd eaten, it's definitely more tedious. But to see that [the food journal] is accessible, to have it on hand all the time, and to be able to enter it as I go along, I find it really practical*.”  - Woman, 19 years old, 10 years living with T1D  *“ I think having [ the coverage vs correction dose] split up is a great way to look at it. Like when I go to like the diabetic nurses, they're always trying to figure out, is it a carb ratio thing, or do we need to adjust the insulin correction factor.”*  - Man, 23 years old, 21 years living with T1D |
| Empowerment | Promoting self-efficacy | Ability for the user to gain control over their T1D management by understanding the impact of various factors (i.e. physical activity, stress levels, sick days, time of insulin injection) on glycemia. | “*If I look at my graph later and see that one day I was trending high all day, but then I look back at the food diary and remember what I ate, I'll understand better*.”  - Woman, 19 years old, 10 years since diagnosis.    “*Let's say one night you take your insulin and [your glucose] spikes, then that's your indication that you need to give insulin earlier. [...] You could say ‘Oh, that's why it went super high versus low’. To me, it could make a big difference.*”  - Woman, 20 years old, 13 years since diagnosis. |
|  | Comprehensive | Encompassing all necessary features deemed useful to users within one app (i.e. note taking, food journal, CGM graph) to improve the practicality of T1D management. | “*I feel like they should integrate automatically. So, for example, there should be a way of taking all that information, [from other apps] , and importing them into this app. But I feel like the app is great and it's s something that I've been waiting for for a long time*”  - Man, 24 years old, 9 years since diagnosis. |
|  | Considerations to reality | App functionalities consider the lived experience of T1D to avoid setting unrealistic expectations. The app incorporates features that considers the day-to-day habits of users (i.e. ability to track whether insulin was administered before or after meals despite the guidelines being to inject before meals). | *“I really liked [the feature of inputting insulin before or after a meal]. I sometimes forget to give myself insulin before I eat. I think it's important to see the trend.”*  - Man, 18 years old, 12 years living with T1D |
|  | Control | The ability to rely on technology while not hindering self-efficacy. This may be hindered by an underlying skepticism of the accuracy of technology-provided information. | “*I’d rather input [the carbohydrates] manually. Especially with the fact that I have to give myself insulin, I don't think it's a good idea to let the AI guess. I'd rather just put in the little effort and just calculate it myself to know exactly how much I need.*”  - Man, 24 years old, 7 years since diagnosis. |
|  | Reassurance | Relating to the security of T1D management (i.e. insulin administration, pump malfunctioning) and ability to validate previous actions (i.e. logging insulin injection) | *"... to have a reminder function, to [remember] to take the long-acting insulin and [the option to] mark it done, because then it just kind of fortifies the idea that, yeah, I did take it and I wouldn't have to go back and check 10 minutes later [to see if I took it]."*  - Man, 24 years old, 9 years since diagnosis. |
| Data verification and override | Human error override | Technology-driven data verification to discern human error. | “*My pump does it for me, but let's say you calculate 44 grams of carbohydrates but you [input it] quickly and add a 0 at the end, it will give you a bolus that is too high. My pump won't let me do this. There needs to be a level of security [in the app]. The app should inform you to check it*.”  - Woman, 19 years old, 7 years living with T1D |
|  | Technology override | Manual user verification and correction of AI generating information. | *“You could choose to accept [the suggestions provided by the AI through photo recognition] or not [...] Like, if it’s wrong, then you still have the option to dismiss it.”*  - Woman, 20 years old, 13 years living with T1D  “*To have the option to enter it manually if you want to override that even if you have a CGM connected. For example, if someone is skeptical about their [CGM] reading and they measure with the typical finger prick and it's a different value, they can input it [manually].*”  - Man, 24 years old, 9 years living with T1D |
