# Supplementary material for: Designing a Carbohydrate Counting App for Young Adults With Type 1 Diabetes: Usability Testing Interview Study
Source: J Med Internet Res. 2026 Mar 31;28:e86024. doi: 10.2196/86024 (PMC13037768; doi:10.2196/86024)
Supplement: Multimedia Appendix 4 [file jmir-v28-e86024-s004.docx]

**Multimedia Appendix 4 - Appreciations and suggestions from the feedback questionnaire after 1 week of app use.**

| App features | Appreciations | Suggestions |
| --- | --- | --- |
| Profile | Appreciation for bilingualism of app (English/French) | Ability to enter multiple correction factors, Remove feature of naming ratios, Ability to input insulin regimen (i.e. basal vs bolus), Personalizing profile (i.e. creating an avatar, adding background photos), Ability to start inputting information and modify later (during the onboarding). |
| Meal entry | Photo recognition facilitates carb counting and insulin dosing, especially for those newly diagnosed with T1D | Indicate that associating tags to meals is optional Prioritize “basic foods” first in database Inaccurate AI-photo recognition, specifically of mixed meals Limited units of measurement (e.g. grams but not millilitre) No added benefit of meal entry when user already knows carbohydrate content of meal. Photo recognition of meals is slow. Add a video tutorial for first time users. |
| Journal | Well organized Clear to see past data and trends Ability to sort for data by events (i.e. exercise) | Adding a notes tab to complement food entry Filter entries by: Date (day, week, month) Characteristic (i.e. insulin requirement) Alphabetical order Manually uploading images is slow and does not accurately detect carbohydrate quantity Add ability to personalize display of journal |
| Graph | Ability to relate blood sugar levels with meals Comprehensive app that includes all features for managing T1D rather than using multiple apps | Color-coordinate different variables on the graph (i.e. fasting blood sugar, target range) Ability to turn phone and see graphic in a landscape view |
| Chatbot | N/A | Add email address or phone number to provide feedback in addition to AI feedback Ability to provide personalized suggestions using data from food journal |
| Suggested Features | Ability to send information (i.e. graph) from app to healthcare professionals Allow the app to be self-improving based on user input Including progress tracking for personal T1D-related goals (i.e. habit streaks) Collaborating with healthcare professionals through the app to provide user-specific recommendations Integrate app with other devices (i.e. smart watches, computers) Ability to earn points with engagement for groceries, travel giveaways, gift cards. Add games related to diabetes. More customizability of the avatar. Ability to link a child’s account to a parent’s version of the app. | |
